# Supplementary material for: Improving Reproducibility to Enhance Scientific Rigor through Consideration of Mouse Diet
Source: Animals (Basel). 2022 Dec 7;12(24):3448. doi: 10.3390/ani12243448 (PMC9774320; doi:10.3390/ani12243448)
Supplement: Supplementary file 1 [file animals-12-03448-s001.zip › animals-2087764-supplementary.pdf]

animals

MDPI

Supplementary Materials

Improving Reproducibility to Enhance Scientific Rigor through Consideration of Mouse Diet

Cara J. Westmark [1,2,*], James Brower [3] and Patrice K. Held[3]

1 Department of Neurology, University of Wisconsin, Madison, WI USA
2 Molecular Environmental Toxicology Center, University of Wisconsin, Madison, WI USA
3 Wisconsin State Laboratory of Hygiene, University of Wisconsin, Madison, WI USA
* Correspondence: westmark@wisc.edu; Tel.: 608-262-9730

Citation: Westmark, C.J.; Brower, J.; Held, P.K. Improving Reproducibility to Enhance Scientific Rigor through Consideration of Mouse Diet. *Animals* **2022**, *12*, 3448. https://doi.org/10.3390/ani12243448

Academic Editor: Domenico Nuzzo

Received: 24 November 2022
Accepted: 5 December 2022
Published: 7 December 2022

Publisher's Note: MDPI stays neutral with regard to jurisdictional claims in published maps and institutional affiliations.

Copyright: © 2022 by the authors. Submitted for possible open access publication under the terms and conditions of the Creative Commons Attribution (CC BY) license (https://creativecommons.org/licenses/by/4.0/).

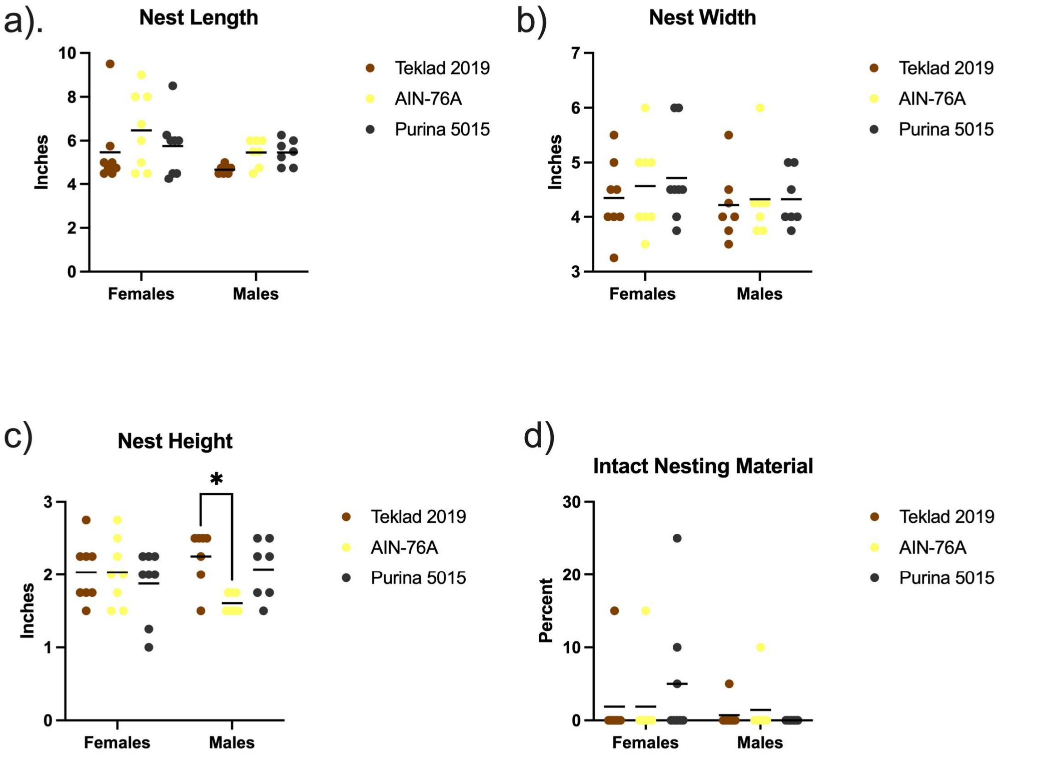

Figure S1. Mouse nesting behavior as a function of diet. Male and female C57BL/6J mice were tested in nest building activity in response to diet (Teklad 2019, AIN-76A or Purina 5015). Overall nest scores are provided in Figure 4 in the text. Here, individual scores for nest length, width and height as well as the percent of intact nesting material after the overnight test are provided. Statistics were determined by 2-way ANOVA and Tukey's multiple comparison tests denoted by $p < 0.05$ (*).

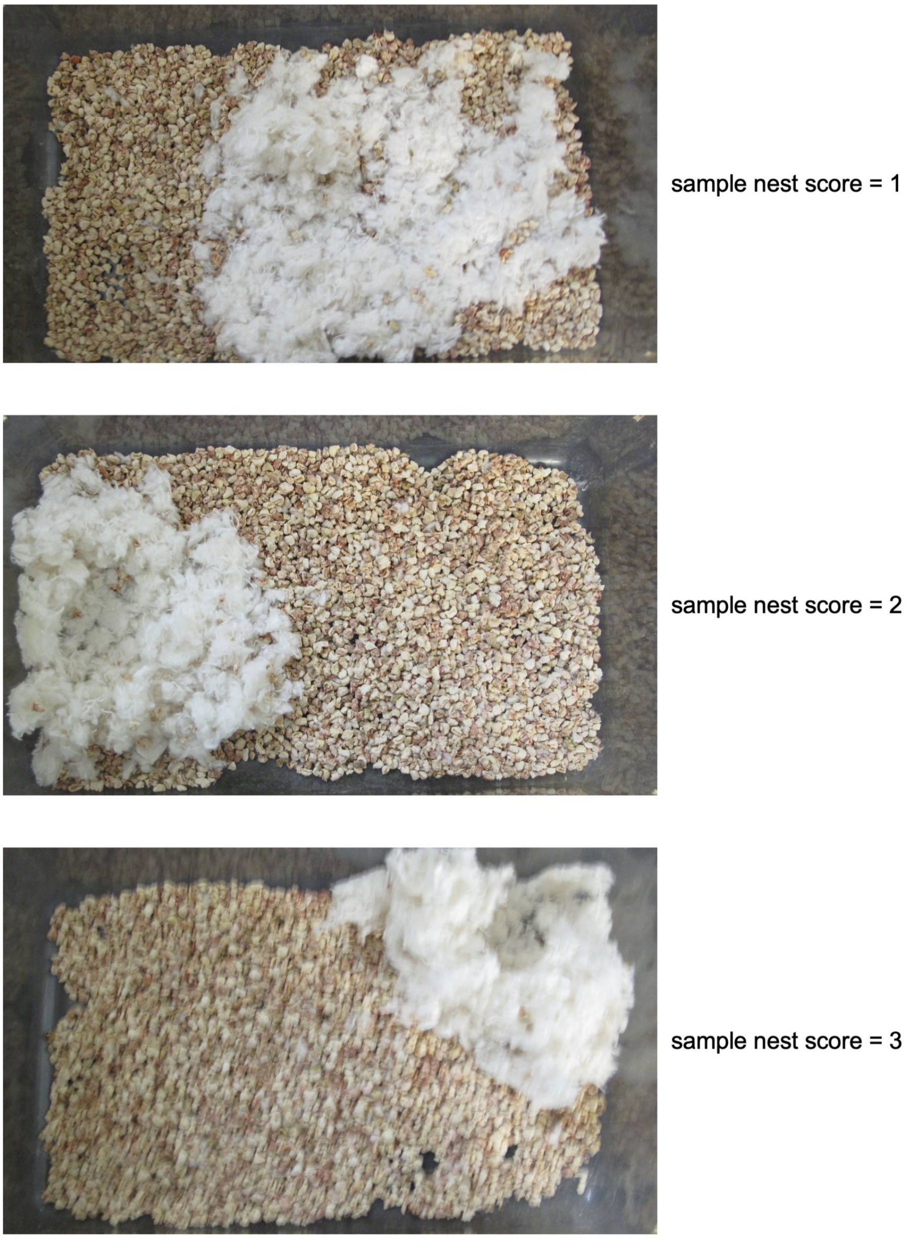

Figure S2. Photographs of mouse nesting behavior as a function of diet. Male and female C57BL/6J mice were tested in nest building activity in response to diet (Teklad 2019, AIN-76A or Purina 5015). Overall nest scores are provided in Figure 4 in the text. Here, representative nests are shown for nest scores of (1) flat nest with partially shredded material, (2) shallow nest with shredded material but lacks fully formed walls, and (3) nest with well-developed walls.

a)

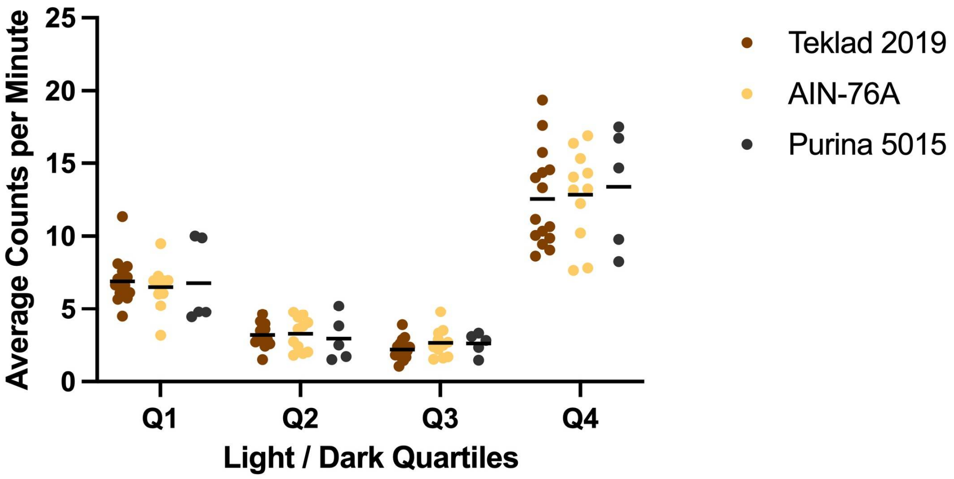

b)

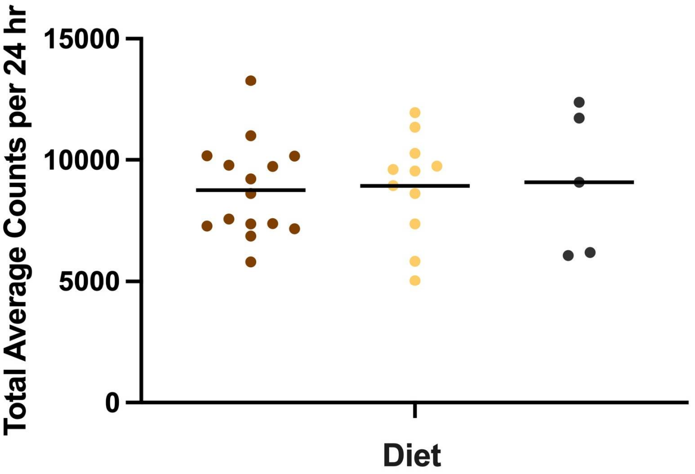

Figure S3. Actigraphy as a function of diet. Data were combined for male and female C57BL/6J mice tested in the actigraphy assay in response to diet (Teklad 2019, AIN-76A or Purina 5015). There was an equipment malfunction during the study resulting in fewer animals per cohort and making it difficult to assess the animals as a function of sex, particularly for the Purina 5015 cohorts. Here, the sexes are combined to increase the n. Data separated by sex are provided in Figure 4cd in the text. Statistics were determined by 2-way ANOVA and Tukey's multiple comparison tests. There were no statistically significant differences. a) average counts per minute for mice were binned into 6-hour quartiles and plotted versus diet. b) total average activity counts per 24-hour period plotted versus diet and sex.

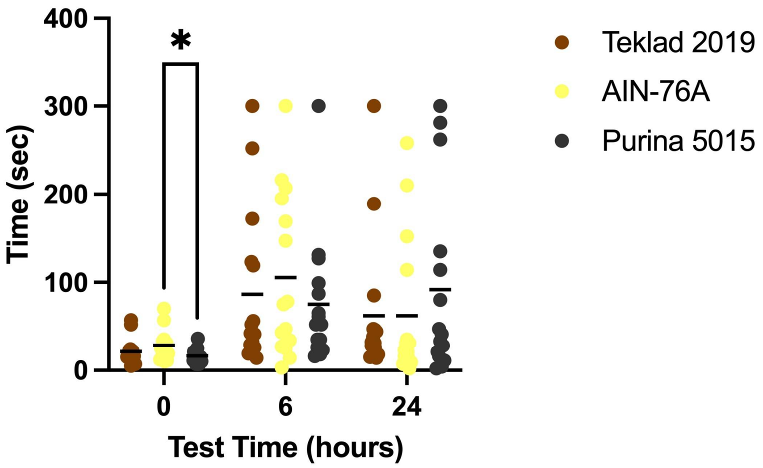

Figure S4. Passive avoidance behavior as a function of diet. Data were combined for male and female C57BL/6J mice tested in the passive avoidance assay in response to diet (Teklad 2019, AIN-76A or Purina 5015). Data separated by sex are provided in Figure 4ef in the text. Statistics were determined by 2-way ANOVA and Tukey's multiple comparison tests. There were no statistically significant differences after the foot shock. Mice fed Purina 5015 had a higher propensity to enter the dark chamber more quickly on the training day. ANOVA results: time x diet $F(4, 84) = 1.058$, $p = 0.38$; time $F(1.991, 83.62) = 15.21$, $p < 0.0001$; diet $F(2, 42) = 0.1007$, $p = 0.90$; and subject $F(42, 84) = 2.334$, $p = 0.0005$. (*) $p < 0.05$.

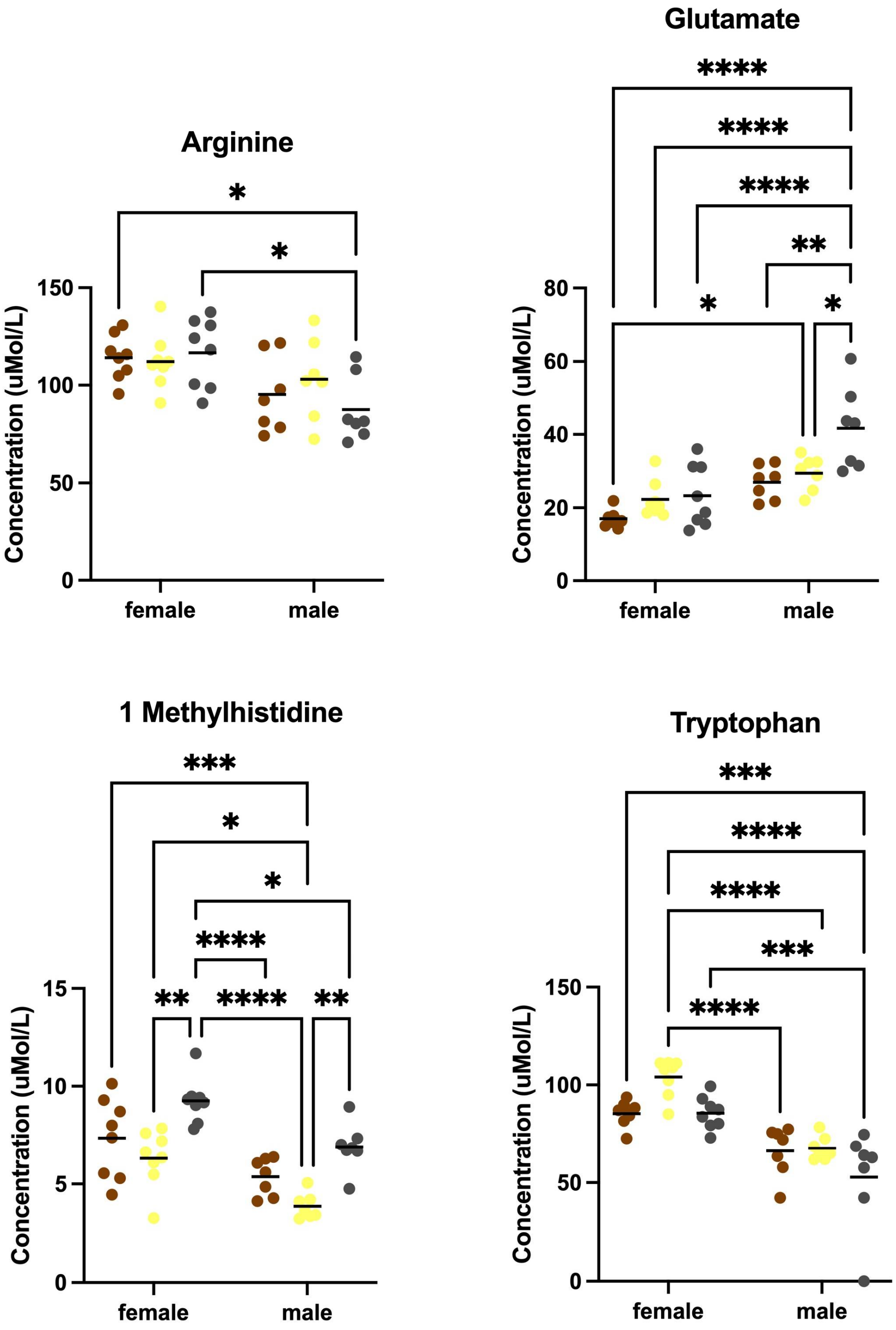

Figure S5. Sex-specific differences in blood plasma amino acid levels as a function of diet. Blood was collected from male and female C57BL/6J mice at euthanization and quantitated for plasma amino acids by ion-exchange chromatography as a function of diet (Teklad 2019, AIN-76A or Purina 5015). Statistics were determined by 2-way ANOVA and Tukey's multiple comparison tests (see Table S1). This figure highlights sex-specific findings. $p < 0.05$ (*), $p < 0.01$ (**), $p < 0.001$ (***) and $p < 0.0001$ (****).

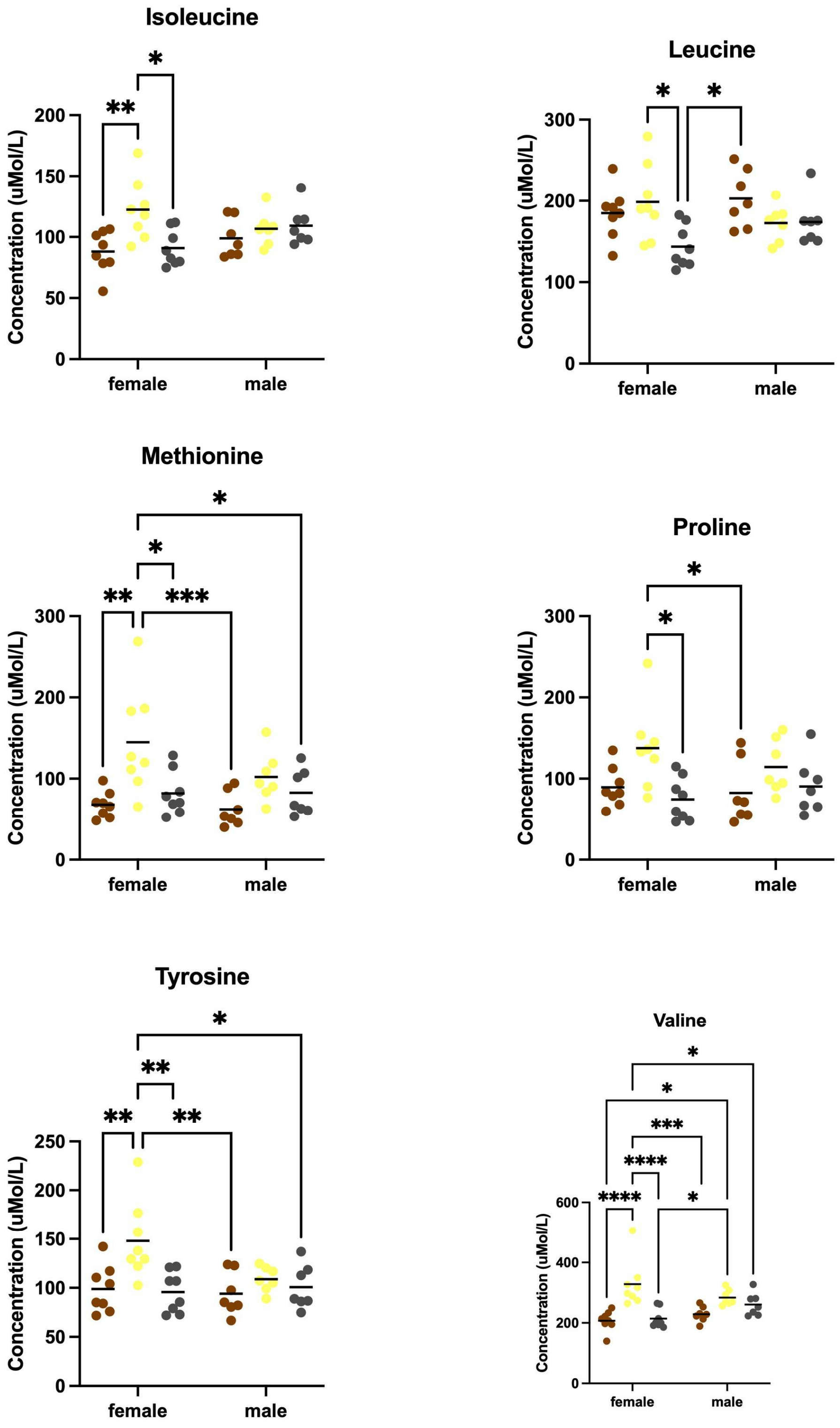

Figure S6. Diet-specific differences in blood plasma amino acid levels. specific to females. Blood was collected from male and female C57BL/6J mice at euthanization and quantitated for plasma amino acids by ion-exchange chromatography as a function of diet (Teklad 2019, AIN-76A or Purina 5015). Statistics were determined by 2-way ANOVA and Tukey's multiple comparison tests (see Table S1). This figure highlights diet-specific differences in females. $p < 0.05$ (*), $p < 0.01$ (**), $p < 0.001$ (***) and $p < 0.0001$ (****).

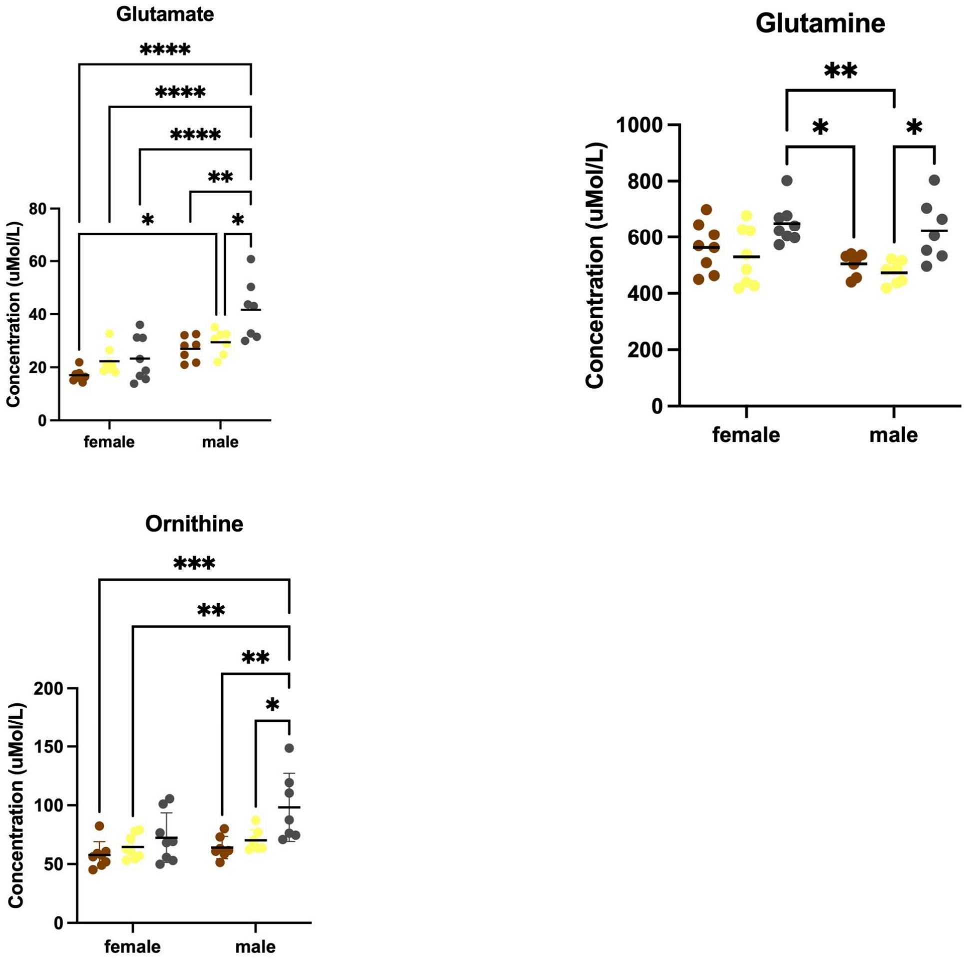

Figure S7. Diet-specific differences in blood plasma amino acid levels specific to males. Blood was collected from male and female C57BL/6J mice at euthanization and quantitated for plasma amino acids by ion-exchange chromatography as a function of diet (Teklad 2019, AIN-76A or Purina 5015). Statistics were determined by 2-way ANOVA and Tukey's multiple comparison tests (see Table S1). This figure highlights diet-specific differences in males. $p < 0.05$ (*), $p < 0.01$ (**), $p < 0.001$ (***) and $p < 0.0001$ (****)

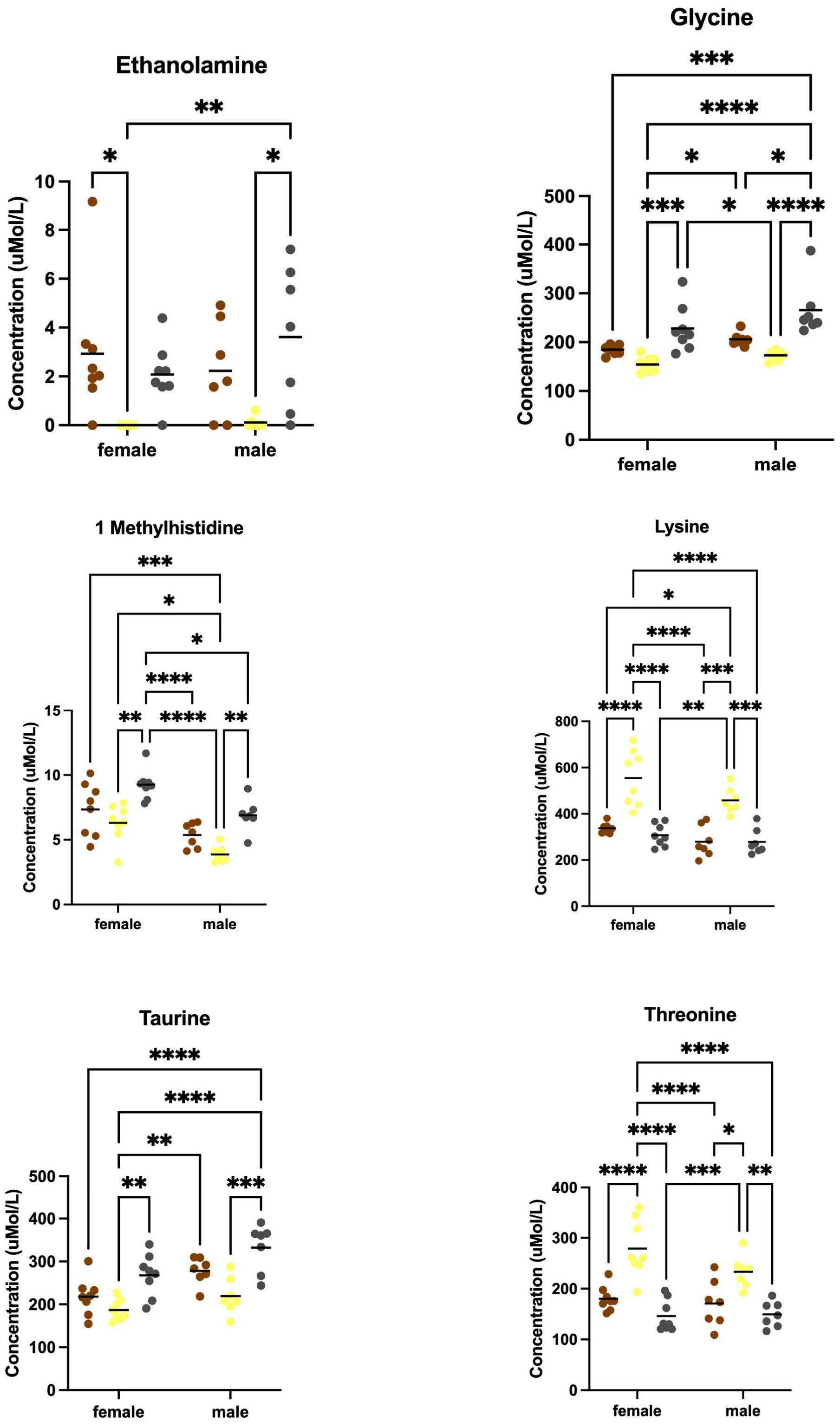

Figure S8. Diet-specific differences in blood plasma amino acid levels in both females and males. Blood was collected from male and female C57BL/6J mice at euthanization and quantitated for plasma amino acids by ion-exchange chromatography as a function of diet (Teklad 2019, AIN-76A or Purina 5015). Statistics were determined by 2-way ANOVA and Tukey's multiple comparison tests (see Table S1). This figure highlights diet-specific differences in both sexes. $p < 0.05$ (*), $p < 0.01$ (**), $p < 0.001$ (***) and $p < 0.0001$ (****).

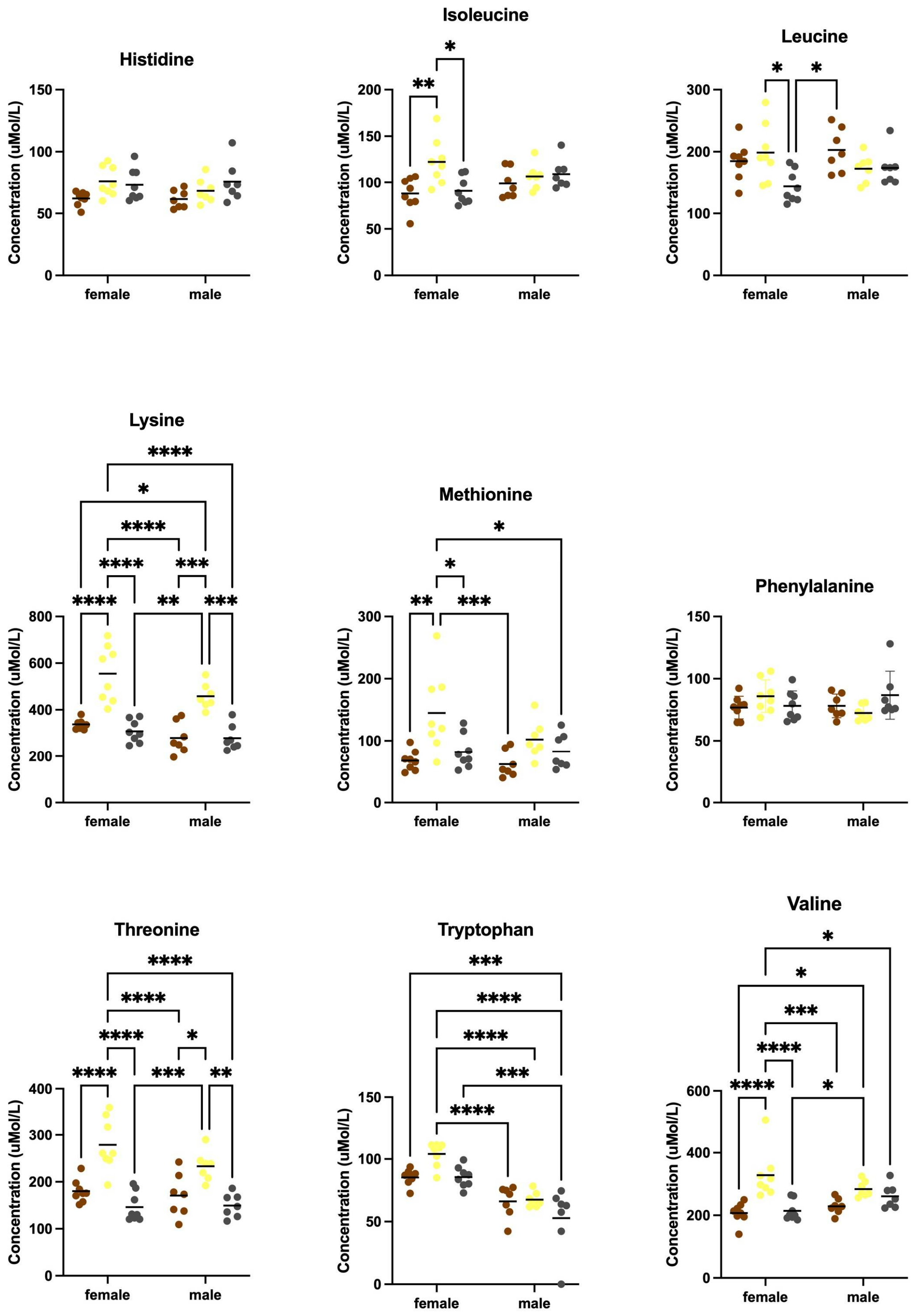

Figure S9. Blood plasma essential amino acid levels as a function of diet. Blood was collected from male and female C57BL/6J mice at euthanization and quantitated for plasma amino acids by ion-exchange chromatography as a function of diet (Teklad 2019, AIN-76A or Purina 5015). Statistics were determined by 2-way ANOVA and Tukey's multiple comparison tests (see Table S1). This figure highlights differences in the nine essential amino acids. $p < 0.05$ (*), $p < 0.01$ (**), $p < 0.001$ (***) and $p < 0.0001$ (****).

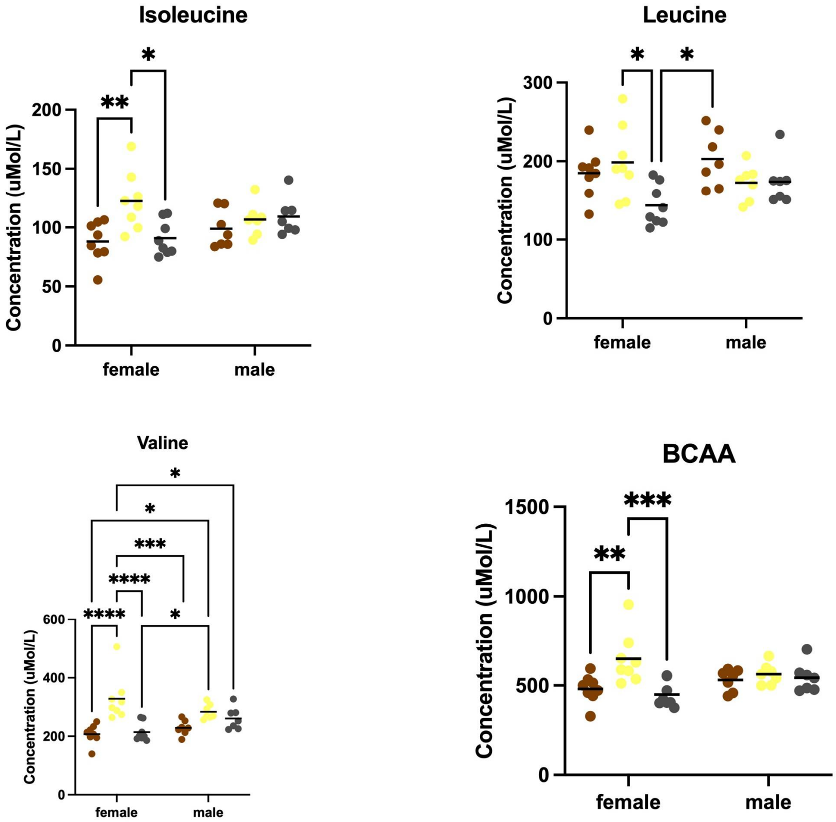

Figure S10. Blood plasma branched chain amino acid levels as a function of diet. Blood was collected from male and female C57BL/6J mice at euthanization and quantitated for plasma amino acids by ion-exchange chromatography as a function of diet (Teklad 2019, AIN-76A or Purina 5015). Statistics were determined by 2-way ANOVA and Tukey's multiple comparison tests (see Table S1). This figure highlights the branched chain amino acids. $p < 0.05$ (*), $p < 0.01$ (**), $p < 0.001$ (***) and $p < 0.0001$ (****).

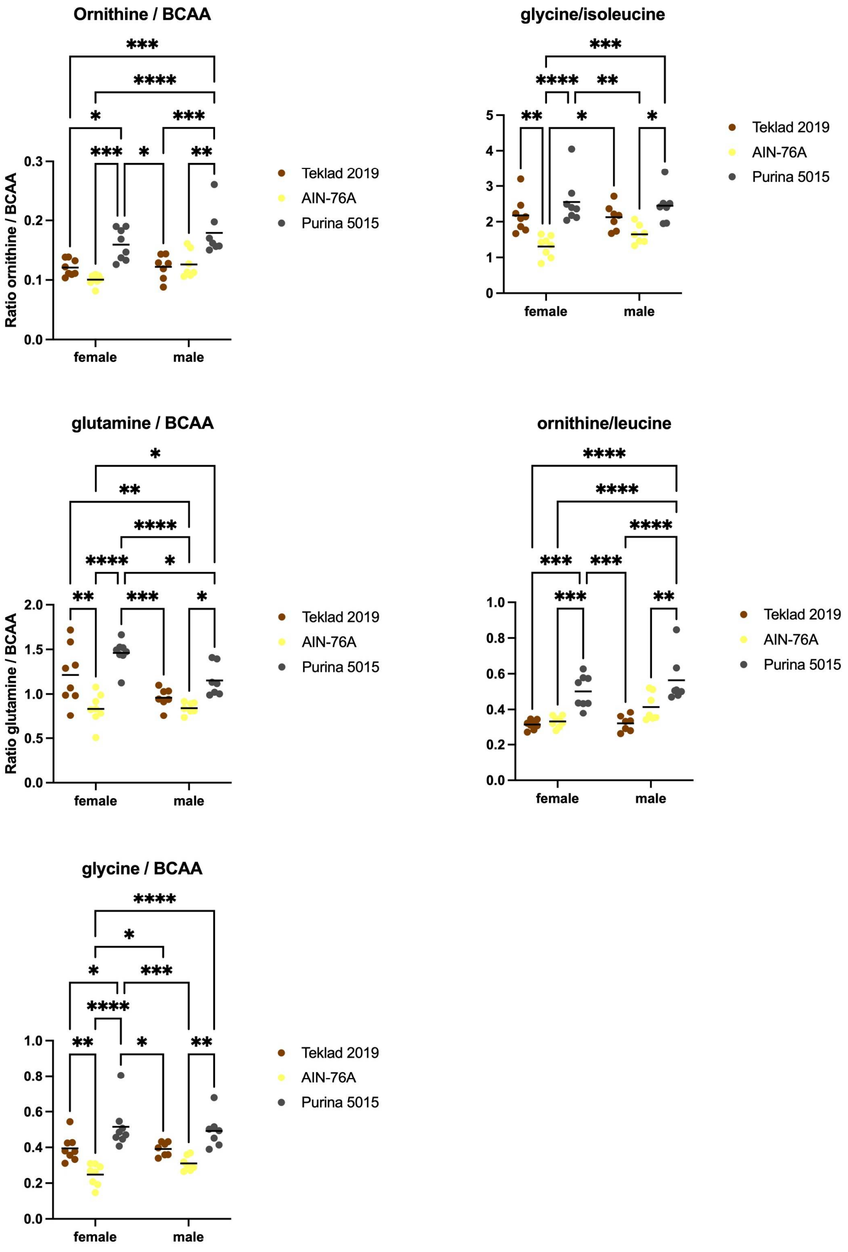

Figure S11. Ratios of blood plasma amino acid levels for ornithine, glutamine and glycine to the BCAAs (isoleucine, leucine and valine) and for glycine:isoleucine and ornithine:leucine as a function of diet. Blood was collected from male and female C57BL/6J mice at euthanization and quantitated for plasma amino acids by ion-exchange chromatography as a function of diet (Teklad 2019, AIN-76A or Purina 5015). Statistics were determined by 2-way ANOVA and Tukey's multiple comparison tests. This figure highlights amino acid ratios previously identified as important in ASD. $p < 0.05$ (*), $p < 0.01$ (**), $p < 0.001$ (***) and $p < 0.0001$ (****).

Table S1. Amino Acid Profile Statistics.

**Amino Acid**	**Females**			**Males**			**Two-Way ANOVA (P value)**		
**Diet**	**2019**	**AIN**	**Purina**	**2019**	**AIN**	**Purina**	**Interaction**	**Diet**	**Sex**
Alanine	451.0	522.4	419.7	399.7	462.2	450.3	0.39	0.15	0.37
β-Alanine	6.36	6.88	6.53	6.89	7.18	6.37	0.92	0.80	0.76
Arginine	114.4	112.3	116.8	95.25	103.1	87.60	0.29	0.67	0.0005
Argininosuccinate	0	0	0	0	0	0	0	0	0
Aspartate	2.45	2.35	2.54	1.94	1.12	1.25	0.52	0.47	0.0021
Carnitine	0	0	0	0	0	0	0	0	0
Citrulline	71.14	73.42	72.83	63.92	70.54	58.60	0.31	0.23	0.010
Cystathionine	0.64	1.25	0.81	0.64	0.95	1.00	0.31	0.023	0.79
Cysteine	0.82	0.25	0.32	0.040	0.10	0.19	0.17	0.42	0.033
Homocysteine	0	0	0	0	0	0	0	0	0
Ethanolamine	2.93	0.0	2.08	2.23	0.11	3.61	0.27	0.0003	0.58
Glutamate	17.03	22.30	23.30	26.96	29.46	41.73	0.067	0.0004	<0.0001
Glutamine	563.2	529.3	648.4	504.4	472.8	622.7	0.82	0.0001	0.058
Glycine	184.6	154.3	228.2	205.9	173.0	265.6	0.68	<0.0001	0.0089
Histidine	62.01	75.75	73.09	61.50	68.23	75.55	0.47	0.0092	0.58
1-Methylhistidine	7.35	6.31	9.25	5.37	3.87	6.90	0.89	<0.0001	<0.0001
3-Methylhistiidine	0.30	0.44	0.0	0.0	0.0	0.0	0.63	0.63	0.20
Isoleucine	88.01	122.4	90.91	98.90	106.7	109.3	0.028	0.0068	0.39
Leucine	184.9	198.7	143.5	202.8	172.6	173.8	0.058	0.014	0.45
Alloisoleucine	0	0	0	0	0	0	0	0	
Lysine	337.5	555.3	307.4	279.0	458.0	278.5	0.40	<0.0001	0.0046
Methionine	67.84	144.9	81.96	62.12	102.2	82.51	0.21	0.0002	0.14
Ornithine	57.81	64.54	72.44	64.12	70.26	98.24	0.19	0.0008	0.016
Phenylalanine	76.81	85.98	78.19	78.17	72.48	86.76	0.052	0.53	0.75
Proline	89.42	137.6	74.45	82.42	114.4	90.27	0.33	0.0026	0.65
Hydroxyproline	11.11	13.78	13.48	16.29	16.23	16.36	0.83	0.83	0.080
Sarcosine	0	0	0	0	0	0	0	0	0
Serine	126.3	146.7	131.9	111.9	129.3	123.5	0.89	0.13	0.082
Anserine	0	0	0	0	0	0	0	0	0
Taurine	218.0	187.0	267.7	278.3	219.3	332.2	0.53	<0.0001	0.0002
Threonine	180.2	278.9	146.3	170.7	233.2	149.4	0.20	<0.0001	0.13
Tryptophan	85.34	104.1	85.60	66.23	67.60	52.78	0.16	0.0041	<0.0001
Tyrosine	98.90	148.0	95.74	94.10	108.9	100.7	0.055	0.0017	0.094
Valine	207.3	328.0	214.3	228.9	283.9	260.6	0.019	<0.0001	0.54
BCAA	480.2	649.1	448.8	530.6	563.3	543.7	0.021	0.0021	0.45
